# A retrospective comparative study of prednisolone use in antagonist co-treated assisted reproductive technology cycles for patients with good prognosis

**DOI:** 10.4274/tjod.12244

**Published:** 2018-09-03

**Authors:** Batuhan Özmen, Emre Göksan Pabuçcu, Yavuz Emre Şükür, Hasan Ulubaşoğlu, Can Ateş, Murat Sönmezer, Bülent Berker, Cem Somer Atabekoğlu

**Affiliations:** 1 Ankara University Faculty of Medicine, Department of Obstetrics and Gynecology, Ankara, Turkey; 2 Ufuk University Faculty of Medicine, Department of Obstetrics and Gynecology, Ankara, Turkey; 3 Ondokuz Mayıs University Faculty of Medicine, Department of Obstetrics and Gynecology, Samsun, Turkey; 4 Ankara University Faculty of Medicine, Department of Biostatistics, Ankara, Turkey

**Keywords:** Assisted reproductive technology, glucocorticoid, gonadotropin-releasing hormone antagonist, peri-implantation period

## Abstract

**Objective:**

To investigate the impact of peri-implantation prednisolone use and its duration in antagonist co-treated assisted reproductive technology (ART) cycles of patients with good prognosis.

**Materials and Methods:**

Infertile patients treated with gonadotropin-releasing hormone antagonist protocol between January 2010 and June 2013 were included. The patients in group A (n=196) received no prednisolone. The patients in groups B (n=397) and C (n=371) received 5 mg oral prednisolone daily, for 4 and 12 days following embryo transfer, respectively. The main outcome parameter was live birth rate.

**Results:**

The ages of the groups were 30.1±4.6, 31.5±4.5, and 30.9±4.7 years, respectively (p=0.163). There was no statistically significant difference between the groups regarding cycle characteristics. Implantation rates were 20.7%, 24.6%, and 23.8%, respectively (p=0.163). Miscarriage rates were 1.5%, 3.5%, and 3.2%, respectively (p=0.859). Live birth rates were 28.7%, 29.3%, and 32.8%, respectively (p=0.482).

**Conclusion:**

Empiric prednisolone administration during the peri-implantation period does not seem to have beneficial effects in ART cycles of patients with good prognosis.

**PRECIS:** In the present study, we evaluated peri-implantation prednisolone use and its duration in a large cohort of patients undergoing assisted reproductive technology with good prognosis.

## Introduction

The implantation process of the embryo is a consequence of complex molecular interactions involving many cytokines, growth factors, and immune cells^(1,2)^. In this regard, several molecules have been suggested to improve implantation and contribute to successful pregnancy when administered during the peri-implantation period. Glucocorticoids, well-known agents with anti-inflammatory and immune suppressive properties, have been investigated for the last few decades and conflicting data have been published^(3,4,5,6,7,8)^. Some authors advocate the beneficial effect in zona-dissected embryos and in the presence of assisted-hatching, whereas others reported significantly higher pregnancies in women with auto-antibodies after the use of glucocorticoids^(6,9,10,11)^. On the contrary, several researchers reported no significant beneficial effect of peri-implantation glucocorticoid administration on implantation and clinical pregnancy rates in intracytoplasmic sperm injection (ICSI) cycles^(12,13)^. Published evidence is too limited and heterogeneous to support any firm conclusion on the value of preimplantation prednisolone use in assisted reproductive technology (ART) for patients considered to have good prognosis. In the present study, we aimed to investigate the impact of peri-implantation prednisolone use and its duration in antagonist co-treated ART cycles of patients with good prognosis.

## Materials and Methods

Infertile patients treated with gonadotropin-releasing hormone (GnRH) antagonist co-treated ART in the Infertility Centre of Ankara University Faculty of Medicine, Turkey, between January 2010 and June 2013 were included in this retrospective cohort study. The clinic where the present study was conducted belongs to a tertiary referral hospital that mainly serves the central and east side of the country with approximately 1000 ART cycles per year. The Institutional Review Board of Ankara University Faculty of Medicine approved the study (approval number: 08-341-16). The first stimulation cycle for each subject was included in the study to prevent possible crossover bias between the groups. The inclusion criteria were being female, age 18-40 years, baseline follicle-stimulating hormone (FSH) level <15 IU/L, diagnosed as tubal factor or unexplained infertility, ICSI treatment, and with complete birth data. The exclusion criteria were body mass index (BMI) >30 kg/m^2^, presence of any untreated thyroid dysfunction/hyperprolactinemia, diminished ovarian reserve according to the Bologna criteria^(14)^ or premature ovarian failure, uterine abnormality, positive tests for antinuclear, anti-double-stranded DNA, anticardiolipin antibodies or lupus anticoagulant, male factor infertility, endometriosis, frozen-thaw cycles, cycles managed with assisted hatching, and cycles with day 5 embryo transfer (ET). Cycle cancellations were performed due to a lack of ovarian response or fertilization failure. For eligible participants, we extracted all data regarding controlled ovarian stimulation (COS) and clinical outcomes from the database, and divided the patients into three groups according to their prednisolone administration protocol. Group A received no prednisolone. Groups B and C received 5 mg oral prednisolone daily for 4 and 12 days following ET, respectively. The different prednisolone protocols were due to the primary physician’s choice. Ovarian stimulation was performed with recombinant FSH (Gonal-F, Merck-Serono, İstanbul, Turkey) beginning from the second day of the menstrual cycle with a fixed starting dosage of 150 IU/day. Dose adjustment was performed individually according to ovarian response. The GnRH antagonist (Cetrotide, Merck-Serono, İstanbul, Turkey) was introduced (0.25 mg/day) on the sixth day (fixed antagonist protocol) and continued throughout ovarian stimulation. When at least three follicles were ≥18 mm, recombinant human chorionic gonadotropin (hCG) 250 µg (Ovitrelle, Merck-Serono, İstanbul, Turkey) was used for final oocyte maturation. Transvaginal ultrasonography-guided oocyte pick-up (OPU) was performed 35-36 hours after the hCG trigger. ET was performed on the 3^rd^ day of OPU. A maximum of two embryos were transferred under ultrasound guidance due to national ET regulations^(15)^. Embryos on the 2^nd^ and 3^rd^ days were classified as cleavage stage embryos and were graded based on cell numbers and the degree of fragmentation. All women were administered luteal phase support through 90 mg/day vaginal micronized progesterone (Crinone 8% gel; Merck-Serono, İstanbul, Turkey) commenced on OPU day. In the event of pregnancy, luteal phase support was continued until 10 weeks of gestation. Pregnancy and clinical pregnancy were defined, respectively, by measuring serum β-hCG levels 2 weeks after ET and as the presence of heartbeat at 6-7 weeks of gestation. The implantation rate was calculated separately for each woman as the number of gestational sacs divided by the number of transferred embryos multiplied by 100. The primary outcome measure was live birth rate (LBR).

### Statistical Analysis

The Statistical Package for the Social Sciences (SPSS Inc., Chicago, IL, United States) 15.0 for Windows software was used for all statistical analyses. The Shapiro-Wilk test was used to test normal distribution of continuous parameters. When distribution of a continuous variable was normal, parametric tests were preferred. Continuous variables were compared using the One-Way ANOVA test. Categorical variables were compared using the chi-square test. Continuous data where descriptive tests used are presented as mean ± standard deviation and categorical data are presented as frequency (percentage). A p value of <0.05 was considered statistically significant. While planning the present study, we were not able to detect any previous studies investigating the effect of peri-implantation period use of prednisolone and its dosage in ART cycles of patients with good prognosis. Hypothetically, when a power analysis was performed with 80% power and an a value of 0.05 for an approximately 5% difference in LBR per cycle, the patient number for each study arm should be 1328 for the confirmation of statistical significance. Thus, in the present study, type 2 statistical error could not be excluded for this parameter. Considering the difficulty of recruiting so many participants to a single-centre trial, the aim was to finish the current trial using the data of the available cohort such that it could be included in future meta-analyses on the issue.

## Results

During the study period, a total of 2970 ART cycles were performed in our unit. Among those, 1226 were first ART cycles of tubal factor or unexplained infertility patients, among whom 78 (6.3%) patients with BMI >30 kg/m^2^ and 184 (15%) patients with frozen-thawed or day 5 ET were excluded. As a result, the data of 964 first ART cycles of patients with good prognosis were found eligible for assessment. 

The demographic characteristics of the study and control groups are presented in Table 1 and the cycle characteristics of the groups are presented in Table 2. The outcome measures of the study are presented in Table 3. There were no statistically significant differences between the groups regarding clinical pregnancy, ongoing pregnancy, and LBRs.

## Discussion

The aim of the present study was to investigate the impact of peri-implantation prednisolone use and its duration in antagonist co-treated ART cycles of patients with good prognosis. We found no significant impact of prednisolone administration during the peri-implantation period and its duration on implantation and clinical pregnancy rates and LBR in antagonist co-treated ART cycles of patients with good prognosis. To the best of our knowledge, this analysis is the largest evaluation of the effect of peri-implantation prednisolone use in antagonist co-treated cycles, and is the only comparison of two different doses of prednisolone. Immune suppressive properties of glucocorticoids have been questioned in terms of enhancing outcomes when administered during peri-implantation period because several factors are effective on implantation process. Although several studies and meta-analyses reported beneficial effects on pregnancy rates, those studies included patients with recurrent miscarriages^(16,17,18)^. However, in our study, we investigated the effect of prednisolone on patients with good prognosis. In the meta-analysis by Boomsma et al.^(7)^ including 14 RCTs and 1879 couples, the empiric use of prednisolone during the peri-implantation period was assessed and a borderline statistically significant increase in pregnancy rates was reported in in vitro fertilization but not in ICSI cycles, suggesting its limited use in ICSI cycles. Despite these results, the authors made their conclusions with caution because the included trials in which ICSI was used were very few and clinically heterogeneous. However, in our study, we included only first ICSI cycles of patients with good prognosis to obtain a relatively homogenous cohort. In the present study, we investigated prednisolone 5 mg because lower doses have already been reported to reveal similar immune-suppression and pregnancy outcomes when compared with higher doses^(12,19,20)^. According to the results of our study, short and long-term use of prednisolone has a similar effect on implantation and pregnancy rates. Additionally, only antagonist co-treated cycles were included because this protocol has widely replaced GnRH agonist cycles globally with its applicability and non-inferior outcomes. The implantation and pregnancy rates were consistent with rates in the available literature, especially with those of Ubaldi et al.^(12)^ who also included patients with good prognosis and used low-dose glucocorticoid. The large number of subjects included in the analyses and the strict inclusion criteria of those with good prognosis were the main strengths of our study. The available LBR data might be of some interest. Moreover, we assessed the impact of prednisolone duration, comparing short and prolonged use. According to the results, neither short nor long-course peri-implantation-period prednisolone administration has any benefit in antagonist co-treated ART cycles of patients with good prognosis. Hence, prednisolone should not be prescribed for routine ART cycles. The results of our study may be used in future meta-analyses investigating prednisolone administration in ART cycles of patients with good prognosis. Large randomized clinical trials may be more suggestive on prednisolone use in patients undergoing ICSI with good prognosis.

The retrospective nature and lack of randomization are the main limitations of the present study. Another limitation is the different manipulations during the COS protocols, mainly dose adjustment and duration of prednisolone treatment, due to primary physician preferences, which could affect the outcome. Moreover, the low power of the statistical analysis can be noted as a limitation. However, given the select nature of our population, attaining such a large cohort was unrealistic in a single-centre study.

## Conclusion

In the present study we could not find an unequivocal beneficial effect of empiric prednisolone administration during the peri-implantation period in women undergoing their first ART cycle with an antagonist protocol. According to the results of our study, within the context of its limitations, a complete shift in clinical practice cannot be suggested.

## Figures and Tables

**Table 1:**
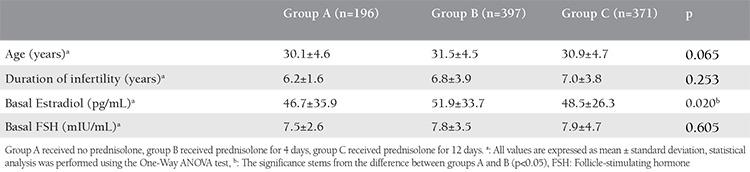
Demographic characteristics of the study groups

**Table 2:**
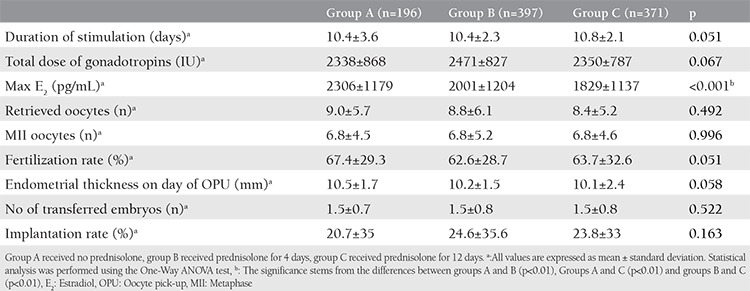
Cycle characteristics of the study groups

**Table 3:**
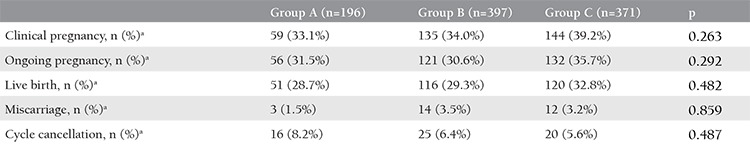
Comparison of outcome measures
